# Supplementary material for: Identification of photoperiod-induced specific miRNAs in the adrenal glands of Sunite sheep (Ovis aries)
Source: Front Vet Sci. 2022 Jul 22;9:888207. doi: 10.3389/fvets.2022.888207 (PMC9354845; doi:10.3389/fvets.2022.888207)
Supplement: Supplementary file 1 [file Data_Sheet_1.ZIP › Supplementary file/miRDeep2 file.zip!/miRDeep2 file/Know_Structure/LP42A3/oar-mir-493.pdf]

miRBase precursor : oar-mir-493
Total read count : 18437
oar-miR-493-5p read count : 13481
oar-miR-493-3p read count : 4955
remaining reads : 1

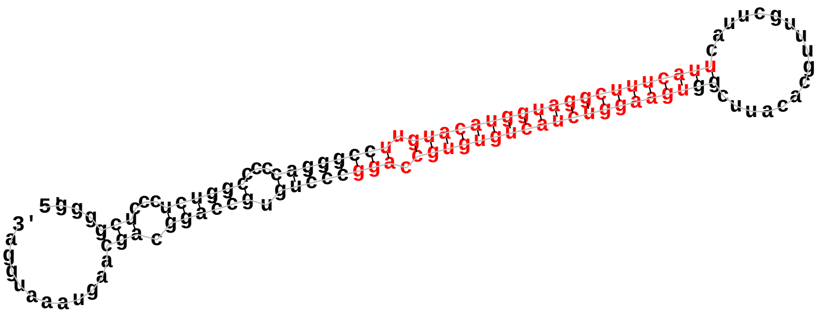

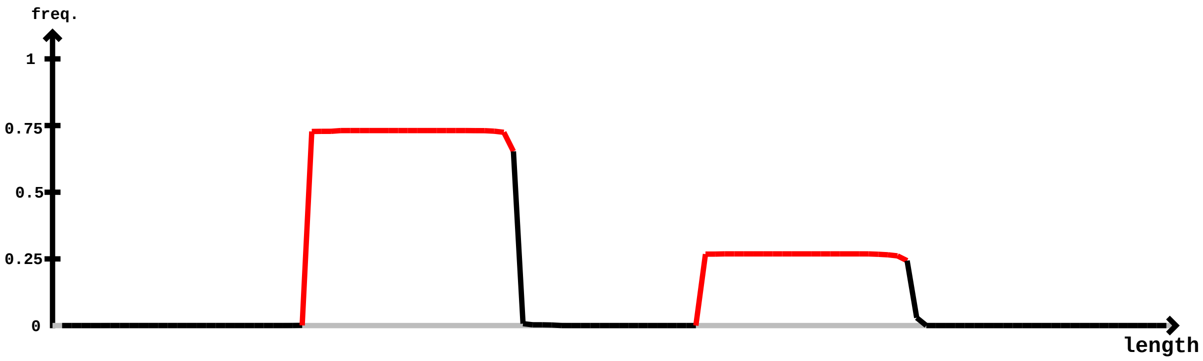

oar-miR-493-5p

oar-miR-493-3p

```
5'- ggggcucccucuggcccccagggccuuguacaugguaggcuuucauucauucguuugcacauucggugaaggucuacugugugccaggcccugugccaggcagcaaguaaaugga  -3'    exp
    ...(((...((((((...((((((((.((((((((((((((((((((.................)))))))))))))))))))).)))))))).)))))).)))...........    reads    mm    sample
    ....cucccucuggcccccagggcc..........................................................................................    1        0     seq
    .......................ccuuguacaugguaggcuuuc.......................................................................    2        0     seq
    .......................ccuuguacaugguaggcuuuca......................................................................    1        0     seq
    ........................cuuguacaugguaggcuuucau.....................................................................    2        0     seq
    ........................cuuguacaugguaggcuuucauu....................................................................    7        0     seq
    .........................uuguacaugguaggcuu.........................................................................    5        0     seq
    .........................uuguacaugguaggcuuu........................................................................    2        0     seq
    .........................uuguacaugguaggcuuuc.......................................................................    33       0     seq
    .........................uuguacaugguaggcuuuca......................................................................    76       0     seq
    .........................uuguacaugguaggcuuucau.....................................................................    1316     0     seq
    .........................uuguacaugguaggcuuucauu....................................................................    11892    0     seq
    .........................uuguacaugguaggcuuucauuc...................................................................    79       0     seq
    .........................uuguacaugguaggcuuucauuca..................................................................    2        0     seq
    .........................uuguacaugguaggcuuucauucauu................................................................    1        0     seq
    ..........................uguacaugguaggcuuucauu....................................................................    10       0     seq
    ...........................guacaugguaggcuuucau.....................................................................    1        0     seq
    ............................uacaugguaggcuuucauu....................................................................    1        0     seq
    ............................uacaugguaggcuuucauuca..................................................................    1        0     seq
    ............................uacaugguaggcuuucauucau.................................................................    13       0     seq
    ............................uacaugguaggcuuucauucauu................................................................    34       0     seq
    ............................uacaugguaggcuuucauucauuc...............................................................    1        0     seq
    .............................acaugguaggcuuucauucauu................................................................    2        0     seq
    ..................................................................ugaaggucuacugugug................................    1        0     seq
    ..................................................................ugaaggucuacugugugU...............................    8        1     seq
    ..................................................................ugaaggucuacugugugc...............................    17       0     seq
    ..................................................................ugaaggucuacugugugcc..............................    38       0     seq
    ..................................................................ugaaggucuacugugugcca.............................    73       0     seq
    ..................................................................ugaaggCcuacugugugcca.............................    1        1     seq
    ..................................................................ugaaggucuacugugugccag............................    327      0     seq
    ..................................................................ugaaggCcuacugugugccag............................    2        1     seq
    ..................................................................ugaaggucuGcugugugccag............................    2        1     seq
    ..................................................................ugaaggucuacuAugugccagg...........................    4        1     seq
    ..................................................................ugaagguUuacugugugccagg...........................    1        1     seq
    ..................................................................ugaaggucuacugugugccagg...........................    3896     0     seq
```

```
                         oar-miR-493-5p
                                                                  oar-miR-493-3p
ggggcucccucuggcccccagggccuuguacaugguaggcuuucauucauucguuugcacauucggugaaggucuacugugugccaggcccugugccaggcagcaaguaaaugga

..................................................................ugaaggucuGcugugugccagg...........................    18    1    seq
..................................................................ugaaUgucuacugugugccagg...........................    4     1    seq
..................................................................ugaaggCcuacugugugccagg...........................    13    1    seq
..................................................................ugaagAucuacugugugccagg...........................    1     1    seq
..................................................................ugaaggucuGcugugugccaggc..........................    3     1    seq
..................................................................ugaaggucuacugugugccaggc..........................    530   0    seq
..................................................................ugaaggCcuacugugugccaggc..........................    1     1    seq
...................................................................gaaggucuacugugugccagg...........................    2     0    seq
....................................................................aaggucuacugugugccagg...........................    7     0    seq
....................................................................aaggucuacugugugccaggc..........................    5     0    seq
....................................................................aaggucuacugugugccaggcc.........................    1     0    seq
```
